# 2,4-Bis(2-eth­oxy­phen­yl)-3-aza­bicyclo­[3.3.1]nonan-9-one

**DOI:** 10.1107/S1600536812044856

**Published:** 2012-11-03

**Authors:** Dong Ho Park, V. Ramkumar, P. Parthiban

**Affiliations:** a Department of Biomedicinal Chemistry, Inje University, Gimhae, Gyeongnam 621 749, Republic of Korea; b Department of Chemistry, IIT Madras, Chennai 600 036, TamilNadu, India

## Abstract

The asymmetric unit of the title compound, C_24_H_29_NO_3_, contains two independent mol­ecules, which each exibit a twin-chair conformation with an equatorial orientation of the *ortho*-eth­oxy­phenyl groups but different dihedral angles [41.3 (1) and 24.1 (1)°] between the benzene rings. In the crystal, pairs of weak C—H⋯O hydrogen bonds link the two different independent mol­ecules into dimers.

## Related literature

For the synthesis and stereochemistry of 3-aza­bicyclo­[3.3.1]nonan-9-ones, see: Park *et al.* (2011 ▶) and for their biological properties, see: Jeyaraman & Avila (1981 ▶); Park *et al.* (2012*a* ▶); Parthiban *et al.* (2010*a* ▶,*b* ▶; 2011*a* ▶). For similar structures, see: Park *et al.* (2012*b* ▶); Parthiban *et al.* (2009*a* ▶,*b* ▶; 2011*b* ▶). For conformational analysis, see: Kalsi (1997 ▶); Cremer & Pople (1975 ▶).
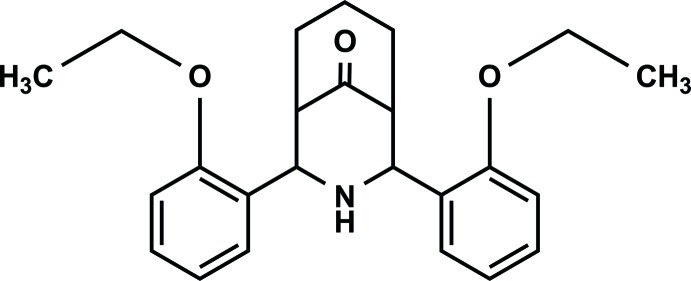


## Experimental

### 

#### Crystal data

C_24_H_29_NO_3_
*M* *_r_* = 379.49
Triclinic, 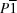

*a* = 9.7981 (3) Å
*b* = 13.6139 (5) Å
*c* = 16.7098 (6) Å
α = 74.363 (2)°
β = 80.464 (2)°
γ = 83.563 (2)°
*V* = 2111.42 (13) Å^3^
*Z* = 4
Mo *K*α radiation
μ = 0.08 mm^−1^
*T* = 298 K
0.35 × 0.28 × 0.22 mm

#### Data collection

Bruker APEXII CCD area-detector diffractometer
Absorption correction: multi-scan (*SADABS*; Bruker, 2004 ▶) *T*_min_ = 0.973, *T*_max_ = 0.983
27579 measured reflections
9839 independent reflections
6037 reflections with *I* > 2σ(*I*)
*R*_int_ = 0.022

#### Refinement

*R*[*F* ^2^ > 2σ(*F* ^2^)] = 0.051
*wR*(*F* ^2^) = 0.150
*S* = 1.02
9839 reflections
509 parameters
H-atom parameters constrained
Δρ_max_ = 0.53 e Å^−3^
Δρ_min_ = −0.45 e Å^−3^

### 

Data collection: *APEX2* (Bruker, 2004 ▶); cell refinement: *SAINT* (Bruker, 2004 ▶); data reduction: *SAINT*; program(s) used to solve structure: *SHELXS97* (Sheldrick, 2008 ▶); program(s) used to refine structure: *SHELXL97* (Sheldrick, 2008 ▶); molecular graphics: *ORTEP-3* (Farrugia, 1997 ▶); software used to prepare material for publication: *SHELXL97*.

## Supplementary Material

Crystal structure: contains datablock(s) global, I. DOI: 10.1107/S1600536812044856/cv5345sup1.cif

Structure factors: contains datablock(s) I. DOI: 10.1107/S1600536812044856/cv5345Isup2.hkl

Supplementary material file. DOI: 10.1107/S1600536812044856/cv5345Isup3.cml

Additional supplementary materials:  crystallographic information; 3D view; checkCIF report

## Figures and Tables

**Table 1:** Hydrogen-bond geometry (Å, °)

*D*—H⋯*A*	*D*—H	H⋯*A*	*D*⋯*A*	*D*—H⋯*A*
C23—H23*A*⋯O1*A*	0.97	2.42	3.311 (3)	153
C23*A*—H23*C*⋯O1	0.97	2.43	3.297 (3)	149

**Fig. 1.**
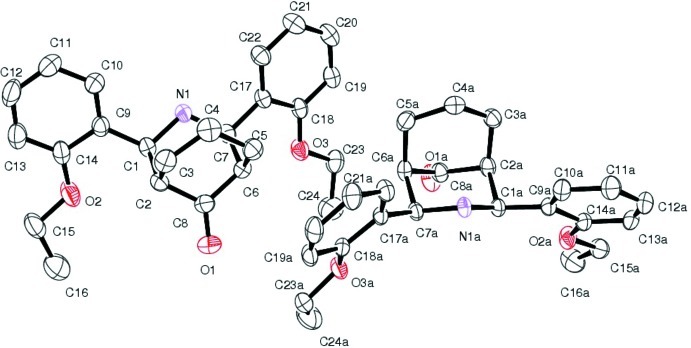
Two independent molecules of the title compound with atomic labels and displacement ellipsoids represented with 30% probability level. H atoms omitted for clarity.

## supplementary crystallographic information

### Comment

Nitrogen containing heterocycles are useful building-blocks of the construction of various pharmacologically important molecules. Since the 3-azabicyclononanes are displying diverse biological actions (Park *et al.*, 2012*a*; Parthiban *et al.*, 2010*a*, 2010*b*, 2011*a*; Jeyaraman & Avila, 1981), and the biological actions mainly depend on the stereochemistry of the molecules, the synthesis as well as stereochemical analysis of any biologically active molecules are of importance in the drug-design and drug-devlopement programs. Based on the above points, we synthesized the title compound, in order to examine the configurational and conformational status by single-crystal X-ray studies.

Careful examination of the asymmery parameters and torsion angles of the title compound reveal that the values are similar to its analogous compounds (Parthiban *et al.*, 2009*a*, 2009*b*, 2011*b*; Park *et al.* 2012*b*). 2,4-Bis(4-ethoxyphenyl)-3-azabicyclo[3.3.1]nonan-9-one is the positional isomer of the title compound that exists in the twin-chair conformation. The impotant torsion angles of the title compound, *viz*., C2—C8—C6—C7 (-58.8 (2)°), C1—C2—C8—C6 (60.8 (2)°), C2—C8—C6—C5 (65.9 (2)°) and C3—C2—C8—C6 (-63.8 (2)°) insist that the bicycle exists in twin-chair conformation. However, the cyclohexanone torsion angles are more deviated than the piperidone ring as well as well the ideal chair cyclohexanone torsion angle of 56° (Kalsi, 1997). The comparision of above with the corresponding torsion angles of the *para*-isomer [-62.5 (2), 62.3 (2), 62.6 (2) and -62.6 (2)°, respectively] indicate that in the title compound, the cyclohexnone ring is more flattened than the cyclohexanone of its *para*-isomer. The above stereochemistry is further witnessed by the Cremer & Pople (1975) ring puckering parameters. For the piperidone ring of the title compound, the total puckering amplitude, Q_T_ is 0.5970 Å and the phase angle θ is 176.66°, for the cyclohexanone, Q_T_ = 0.5590 Å and θ = 163.39°. The same for the *para*-isomer are, Q_T_ = 0.5999 Å and θ = 173.84° (piperidone) and Q_T_ = 0.5643 Å and θ = 168.44°(cyclohexanone). Further, the orientation of the ethoxyphenyl groups on both sides of the secondary amino group are identified by their torsion angles. The torsion angle of C8—C2—C1—C9 and C8—C6—C7—C17 are 176.24 (15) and -179.42 (15)°, respectively.

The two benzene rings in two independent molecules are inclined to each other with angles of 41.3 (1) and 24.1 (1)°, respectively. In the crystal, weak intermolecular C—H···O interactions (Table 1) link independent molecules into dimer.

### Experimental

The 2,4-*bis*(2-ethoxyphenyl)-3-azabicyclo[3.3.1]nonan-9-one was synthesized by a modified and an optimized double Mannich condensation in one-pot, using 2-ethoxybenzaldehyde (0.1 mol, 15.018 g/13.94 ml), cyclohexanone (0.05 mol, 4.90 g/5.18 ml) and ammonium acetate (0.075 mol, 5.78 g) in a 50 ml of absolute ethanol (Park *et al.*, 2011). The mixture was gently warmed on a hot plate at 303–308 K (30–35° C) with moderate stirring till the complete consumption of the starting materials, which was monitored by TLC. After completion of the rection, the crude compound was separated by filtration and gently washed with 1:5 cold ethanol-ether mixture. X-ray diffraction quality crystals of the title compound were obtained by slow evaporation from ethanol.

### Refinement

All hydrogen atoms were fixed geometrically and allowed to ride on the parent carbon atoms, with C—H = 0.93–0.98 Å and N—H = 0.86 Å, and with *U*_iso_(H) = 1.2–1.5*U*_eq_ of the parent atom.

### Crystal data

**Table:**

C_24_H_29_NO_3_	*Z* = 4
*M**_r_* = 379.49	*F*(000) = 816
Triclinic, *P*1	*D*_x_ = 1.194 Mg m^−^^3^
Hall symbol: -P 1	Mo *K*α radiation, λ = 0.71073 Å
*a* = 9.7981 (3) Å	Cell parameters from 5334 reflections
*b* = 13.6139 (5) Å	θ = 0.0–0.0°
*c* = 16.7098 (6) Å	µ = 0.08 mm^−^^1^
α = 74.363 (2)°	*T* = 298 K
β = 80.464 (2)°	Block, colourless
γ = 83.563 (2)°	0.35 × 0.28 × 0.22 mm
*V* = 2111.42 (13) Å^3^	

### Data collection

**Table:**

Bruker APEXII CCD area-detector diffractometer	9839 independent reflections
Radiation source: fine-focus sealed tube	6037 reflections with *I* > 2σ(*I*)
Graphite monochromator	*R*_int_ = 0.022
phi and ω scans	θ_max_ = 28.6°, θ_min_ = 2.1°
Absorption correction: multi-scan (*SADABS*; Bruker, 2004)	*h* = −12→12
*T*_min_ = 0.973, *T*_max_ = 0.983	*k* = −17→18
27579 measured reflections	*l* = −21→22

### Refinement

**Table:**

Refinement on *F*^2^	Primary atom site location: structure-invariant direct methods
Least-squares matrix: full	Secondary atom site location: difference Fourier map
*R*[*F*^2^ > 2σ(*F*^2^)] = 0.051	Hydrogen site location: inferred from neighbouring sites
*wR*(*F*^2^) = 0.150	H-atom parameters constrained
*S* = 1.02	*w* = 1/[σ^2^(*F*_o_^2^) + (0.0546*P*)^2^ + 0.6801*P*] where *P* = (*F*_o_^2^ + 2*F*_c_^2^)/3
9839 reflections	(Δ/σ)_max_ < 0.001
509 parameters	Δρ_max_ = 0.53 e Å^−^^3^
0 restraints	Δρ_min_ = −0.45 e Å^−^^3^

### Special details

**Table:**

Geometry. All e.s.d.'s (except the e.s.d. in the dihedral angle between two l.s. planes)are estimated using the full covariance matrix. The cell e.s.d.'s are takeninto account individually in the estimation of e.s.d.'s in distances, anglesand torsion angles; correlations between e.s.d.'s in cell parameters are onlyused when they are defined by crystal symmetry. An approximate (isotropic)treatment of cell e.s.d.'s is used for estimating e.s.d.'s involving l.s. planes.
Refinement. Refinement of *F*^2^ against ALL reflections. The weighted *R*-factor *wR* and goodness of fit *S* are based on *F*^2^, conventional *R*-factors *R* are based on *F*, with *F* set to zero for negative *F*^2^. The threshold expression of *F*^2^ > σ(*F*^2^) is used only for calculating *R*-factors(gt) *etc*. and is not relevant to the choice of reflections for refinement. *R*-factors based on *F*^2^ are statistically about twice as large as those based on *F*, and *R*- factors based on ALL data will be even larger.

### Fractional atomic coordinates and isotropic or equivalent isotropic displacement parameters (Å^2^)

**Table:**

	*x*	*y*	*z*	*U*_iso_*/*U*_eq_	
C1	0.36743 (18)	0.85897 (14)	0.06351 (11)	0.0500 (4)	
H1	0.4178	0.8488	0.0103	0.060*	
C2	0.25640 (19)	0.77992 (14)	0.09612 (12)	0.0533 (4)	
H2	0.1937	0.7901	0.0541	0.064*	
C3	0.1701 (2)	0.78294 (16)	0.18078 (13)	0.0647 (5)	
H3A	0.1321	0.8526	0.1778	0.078*	
H3B	0.0927	0.7406	0.1902	0.078*	
C4	0.2490 (2)	0.74703 (17)	0.25537 (13)	0.0695 (6)	
H4A	0.3019	0.8022	0.2574	0.083*	
H4B	0.1828	0.7322	0.3063	0.083*	
C5	0.3472 (2)	0.65312 (15)	0.25377 (13)	0.0616 (5)	
H5A	0.2940	0.5931	0.2713	0.074*	
H5B	0.4107	0.6462	0.2941	0.074*	
C6	0.4315 (2)	0.65624 (14)	0.16746 (12)	0.0536 (5)	
H6	0.4811	0.5892	0.1692	0.064*	
C7	0.53772 (18)	0.73929 (14)	0.13630 (11)	0.0488 (4)	
H7	0.5881	0.7323	0.0821	0.059*	
C8	0.3305 (2)	0.67594 (15)	0.10576 (13)	0.0586 (5)	
C9	0.29786 (19)	0.96590 (14)	0.04698 (13)	0.0554 (5)	
C10	0.2861 (2)	1.02513 (16)	0.10223 (16)	0.0747 (6)	
H10	0.3272	1.0012	0.1508	0.090*	
C11	0.2138 (3)	1.12062 (19)	0.0872 (2)	0.0993 (9)	
H11	0.2060	1.1604	0.1253	0.119*	
C12	0.1538 (3)	1.15537 (18)	0.0147 (2)	0.0938 (9)	
H12	0.1057	1.2194	0.0039	0.113*	
C13	0.1635 (2)	1.09799 (18)	−0.04144 (17)	0.0791 (7)	
H13	0.1215	1.1224	−0.0897	0.095*	
C14	0.2359 (2)	1.00353 (16)	−0.02670 (14)	0.0635 (5)	
C15	0.1763 (3)	0.9619 (2)	−0.14731 (16)	0.0920 (8)	
H15A	0.2042	1.0245	−0.1879	0.110*	
H15B	0.0777	0.9700	−0.1282	0.110*	
C16	0.2073 (4)	0.8738 (3)	−0.1860 (2)	0.1269 (12)	
H16A	0.3050	0.8670	−0.2050	0.190*	
H16B	0.1571	0.8854	−0.2327	0.190*	
H16C	0.1799	0.8123	−0.1451	0.190*	
C17	0.64188 (18)	0.72424 (14)	0.19648 (11)	0.0486 (4)	
C18	0.75334 (18)	0.65048 (14)	0.19377 (11)	0.0505 (4)	
C19	0.8500 (2)	0.63614 (16)	0.24840 (12)	0.0581 (5)	
H19	0.9245	0.5878	0.2460	0.070*	
C20	0.8364 (2)	0.69305 (18)	0.30616 (13)	0.0651 (6)	
H20	0.9017	0.6828	0.3427	0.078*	
C21	0.7274 (2)	0.76473 (18)	0.31044 (13)	0.0671 (6)	
H21	0.7179	0.8028	0.3499	0.081*	
C22	0.6315 (2)	0.77980 (16)	0.25514 (12)	0.0585 (5)	
H22	0.5580	0.8289	0.2578	0.070*	
C23	0.8671 (2)	0.51955 (16)	0.12899 (14)	0.0691 (6)	
H23A	0.8744	0.4733	0.1838	0.083*	
H23B	0.9548	0.5504	0.1076	0.083*	
C24	0.8344 (3)	0.4629 (2)	0.0709 (2)	0.1067 (10)	
H24A	0.7482	0.4319	0.0930	0.160*	
H24B	0.9071	0.4108	0.0650	0.160*	
H24C	0.8267	0.5094	0.0170	0.160*	
C1A	0.64687 (16)	0.15039 (12)	0.44479 (10)	0.0422 (4)	
H1A	0.6561	0.1204	0.3968	0.051*	
C2A	0.76566 (17)	0.22198 (13)	0.42932 (11)	0.0473 (4)	
H2A	0.8545	0.1818	0.4231	0.057*	
C3A	0.7659 (2)	0.27739 (14)	0.49824 (12)	0.0556 (5)	
H3A1	0.7619	0.2270	0.5520	0.067*	
H3A2	0.8529	0.3093	0.4883	0.067*	
C4A	0.6469 (2)	0.35883 (15)	0.50361 (12)	0.0602 (5)	
H4A1	0.6689	0.4024	0.5364	0.072*	
H4A2	0.5636	0.3256	0.5329	0.072*	
C5A	0.6178 (2)	0.42519 (14)	0.41788 (13)	0.0589 (5)	
H5A1	0.6878	0.4745	0.3966	0.071*	
H5A2	0.5285	0.4630	0.4247	0.071*	
C6A	0.61683 (18)	0.36447 (13)	0.35247 (11)	0.0484 (4)	
H6A	0.6112	0.4128	0.2976	0.058*	
C7A	0.50066 (17)	0.28949 (12)	0.37073 (10)	0.0426 (4)	
H7A	0.5136	0.2557	0.3248	0.051*	
C8A	0.75265 (18)	0.30110 (14)	0.34832 (12)	0.0496 (4)	
C9A	0.65574 (18)	0.06396 (12)	0.52316 (11)	0.0450 (4)	
C10A	0.5745 (2)	0.06544 (14)	0.59797 (12)	0.0576 (5)	
H10A	0.5097	0.1203	0.6007	0.069*	
C11A	0.5869 (3)	−0.01343 (16)	0.66985 (13)	0.0712 (6)	
H11A	0.5304	−0.0116	0.7200	0.085*	
C12A	0.6830 (3)	−0.09377 (15)	0.66610 (15)	0.0729 (6)	
H12A	0.6926	−0.1461	0.7143	0.087*	
C13A	0.7656 (2)	−0.09789 (14)	0.59183 (15)	0.0638 (6)	
H13A	0.8301	−0.1531	0.5898	0.077*	
C14A	0.75241 (19)	−0.01967 (12)	0.52014 (12)	0.0503 (4)	
C15A	0.9384 (2)	−0.09232 (16)	0.43565 (16)	0.0736 (7)	
H15C	0.9027	−0.1594	0.4497	0.088*	
H15D	1.0042	−0.0933	0.4734	0.088*	
C16A	1.0074 (3)	−0.0658 (2)	0.34722 (17)	0.0906 (8)	
H16D	0.9444	−0.0723	0.3109	0.136*	
H16E	1.0885	−0.1114	0.3413	0.136*	
H16F	1.0339	0.0033	0.3323	0.136*	
C17A	0.35634 (17)	0.34232 (12)	0.37438 (10)	0.0419 (4)	
C18A	0.28975 (18)	0.36874 (12)	0.30268 (11)	0.0451 (4)	
C19A	0.15377 (19)	0.41012 (14)	0.30671 (13)	0.0563 (5)	
H19A	0.1086	0.4249	0.2596	0.068*	
C20A	0.0859 (2)	0.42923 (15)	0.37999 (14)	0.0643 (6)	
H20A	−0.0052	0.4574	0.3822	0.077*	
C21A	0.1504 (2)	0.40738 (16)	0.45031 (13)	0.0637 (5)	
H21A	0.1046	0.4224	0.4995	0.076*	
C22A	0.28471 (19)	0.36263 (14)	0.44692 (12)	0.0538 (5)	
H22A	0.3276	0.3459	0.4950	0.065*	
C23A	0.3037 (2)	0.36747 (16)	0.15767 (12)	0.0666 (6)	
H23C	0.2675	0.4381	0.1408	0.080*	
H23D	0.2278	0.3237	0.1677	0.080*	
C24A	0.4124 (3)	0.3436 (2)	0.09121 (15)	0.1005 (9)	
H24D	0.4836	0.3907	0.0790	0.151*	
H24E	0.3719	0.3496	0.0414	0.151*	
H24F	0.4520	0.2751	0.1102	0.151*	
N1	0.46630 (15)	0.84156 (11)	0.12323 (9)	0.0500 (4)	
H1B	0.4809	0.8864	0.1480	0.060*	
N1A	0.51362 (13)	0.21006 (10)	0.44872 (8)	0.0422 (3)	
H1A1	0.4502	0.1998	0.4917	0.051*	
O1	0.3081 (2)	0.61501 (12)	0.06929 (11)	0.0909 (5)	
O2	0.25195 (18)	0.94051 (14)	−0.07861 (10)	0.0878 (5)	
O3	0.75755 (14)	0.59694 (11)	0.13539 (9)	0.0661 (4)	
O1A	0.84094 (15)	0.31195 (12)	0.28738 (9)	0.0741 (4)	
O2A	0.82815 (14)	−0.01692 (10)	0.44357 (9)	0.0644 (4)	
O3A	0.36692 (14)	0.35032 (10)	0.23189 (8)	0.0589 (3)	

### Atomic displacement parameters (Å^2^)

**Table:**

	*U*^11^	*U*^22^	*U*^33^	*U*^12^	*U*^13^	*U*^23^
C1	0.0456 (10)	0.0543 (10)	0.0472 (10)	−0.0013 (8)	−0.0110 (8)	−0.0063 (8)
C2	0.0506 (10)	0.0540 (11)	0.0582 (11)	−0.0054 (8)	−0.0189 (9)	−0.0116 (9)
C3	0.0476 (11)	0.0613 (12)	0.0793 (15)	−0.0091 (9)	−0.0014 (10)	−0.0105 (11)
C4	0.0655 (13)	0.0780 (14)	0.0604 (13)	−0.0117 (11)	0.0062 (10)	−0.0165 (11)
C5	0.0645 (12)	0.0567 (11)	0.0587 (12)	−0.0146 (10)	−0.0085 (10)	−0.0025 (9)
C6	0.0604 (11)	0.0431 (9)	0.0590 (11)	0.0024 (8)	−0.0152 (9)	−0.0143 (8)
C7	0.0473 (10)	0.0551 (10)	0.0418 (9)	0.0023 (8)	−0.0075 (8)	−0.0106 (8)
C8	0.0666 (13)	0.0530 (11)	0.0600 (12)	−0.0098 (9)	−0.0135 (10)	−0.0162 (9)
C9	0.0470 (10)	0.0486 (10)	0.0663 (12)	−0.0060 (8)	−0.0142 (9)	−0.0028 (9)
C10	0.0813 (15)	0.0523 (12)	0.0957 (17)	−0.0022 (11)	−0.0362 (13)	−0.0146 (12)
C11	0.116 (2)	0.0553 (14)	0.139 (3)	0.0024 (14)	−0.049 (2)	−0.0305 (15)
C12	0.0855 (18)	0.0458 (12)	0.140 (3)	0.0024 (12)	−0.0352 (17)	0.0019 (15)
C13	0.0705 (15)	0.0619 (14)	0.0921 (18)	−0.0055 (11)	−0.0250 (13)	0.0106 (13)
C14	0.0512 (11)	0.0592 (12)	0.0702 (14)	−0.0024 (9)	−0.0175 (10)	0.0052 (11)
C15	0.0973 (19)	0.0965 (19)	0.0769 (16)	−0.0138 (15)	−0.0428 (14)	0.0078 (14)
C16	0.162 (3)	0.133 (3)	0.100 (2)	0.015 (2)	−0.057 (2)	−0.041 (2)
C17	0.0442 (10)	0.0558 (10)	0.0414 (9)	−0.0019 (8)	−0.0066 (7)	−0.0052 (8)
C18	0.0470 (10)	0.0554 (10)	0.0417 (10)	−0.0013 (8)	−0.0052 (8)	−0.0015 (8)
C19	0.0463 (10)	0.0652 (12)	0.0538 (11)	0.0013 (9)	−0.0092 (9)	−0.0010 (10)
C20	0.0511 (12)	0.0883 (15)	0.0521 (12)	−0.0092 (11)	−0.0147 (9)	−0.0056 (11)
C21	0.0571 (12)	0.0916 (16)	0.0579 (12)	−0.0059 (11)	−0.0111 (10)	−0.0261 (11)
C22	0.0492 (11)	0.0708 (13)	0.0559 (11)	−0.0002 (9)	−0.0092 (9)	−0.0172 (10)
C23	0.0626 (13)	0.0655 (13)	0.0713 (14)	0.0150 (10)	−0.0071 (11)	−0.0133 (11)
C24	0.108 (2)	0.103 (2)	0.124 (2)	0.0248 (17)	−0.0270 (19)	−0.0614 (19)
C1A	0.0416 (9)	0.0383 (8)	0.0458 (9)	0.0075 (7)	−0.0142 (7)	−0.0083 (7)
C2A	0.0358 (9)	0.0492 (10)	0.0518 (10)	0.0056 (7)	−0.0110 (7)	−0.0050 (8)
C3A	0.0541 (11)	0.0552 (11)	0.0580 (11)	−0.0090 (9)	−0.0212 (9)	−0.0052 (9)
C4A	0.0675 (13)	0.0555 (11)	0.0641 (13)	−0.0064 (10)	−0.0174 (10)	−0.0208 (10)
C5A	0.0560 (11)	0.0425 (10)	0.0771 (14)	−0.0019 (8)	−0.0147 (10)	−0.0108 (9)
C6A	0.0466 (10)	0.0443 (9)	0.0452 (10)	0.0034 (8)	−0.0100 (8)	0.0037 (8)
C7A	0.0428 (9)	0.0427 (9)	0.0412 (9)	0.0071 (7)	−0.0144 (7)	−0.0079 (7)
C8A	0.0421 (10)	0.0531 (10)	0.0502 (11)	−0.0038 (8)	−0.0078 (8)	−0.0064 (8)
C9A	0.0457 (9)	0.0381 (9)	0.0504 (10)	0.0002 (7)	−0.0169 (8)	−0.0054 (7)
C10A	0.0653 (12)	0.0477 (10)	0.0550 (12)	0.0000 (9)	−0.0130 (10)	−0.0038 (9)
C11A	0.0909 (16)	0.0619 (13)	0.0550 (12)	−0.0117 (12)	−0.0132 (11)	−0.0008 (10)
C12A	0.1019 (18)	0.0451 (11)	0.0682 (15)	−0.0135 (12)	−0.0378 (13)	0.0100 (10)
C13A	0.0768 (14)	0.0348 (9)	0.0820 (15)	0.0025 (9)	−0.0395 (12)	−0.0045 (10)
C14A	0.0511 (10)	0.0382 (9)	0.0642 (12)	0.0023 (8)	−0.0258 (9)	−0.0091 (8)
C15A	0.0755 (14)	0.0576 (12)	0.1014 (18)	0.0314 (11)	−0.0464 (13)	−0.0378 (12)
C16A	0.0789 (16)	0.105 (2)	0.100 (2)	0.0374 (15)	−0.0292 (15)	−0.0553 (16)
C17A	0.0396 (9)	0.0363 (8)	0.0480 (10)	0.0039 (7)	−0.0129 (7)	−0.0064 (7)
C18A	0.0473 (10)	0.0348 (8)	0.0513 (10)	0.0008 (7)	−0.0163 (8)	−0.0036 (7)
C19A	0.0483 (11)	0.0501 (10)	0.0670 (13)	0.0043 (8)	−0.0256 (10)	−0.0016 (9)
C20A	0.0410 (10)	0.0598 (12)	0.0801 (15)	0.0105 (9)	−0.0118 (10)	−0.0011 (11)
C21A	0.0517 (11)	0.0672 (13)	0.0613 (12)	0.0137 (10)	−0.0016 (9)	−0.0089 (10)
C22A	0.0496 (10)	0.0565 (11)	0.0526 (11)	0.0085 (9)	−0.0146 (9)	−0.0100 (9)
C23A	0.0905 (16)	0.0575 (12)	0.0560 (12)	0.0004 (11)	−0.0338 (11)	−0.0100 (10)
C24A	0.122 (2)	0.126 (2)	0.0593 (15)	0.0061 (19)	−0.0238 (15)	−0.0328 (15)
N1	0.0488 (8)	0.0465 (8)	0.0555 (9)	−0.0023 (7)	−0.0180 (7)	−0.0085 (7)
N1A	0.0365 (7)	0.0422 (7)	0.0420 (8)	0.0067 (6)	−0.0079 (6)	−0.0033 (6)
O1	0.1271 (15)	0.0672 (10)	0.0981 (12)	−0.0053 (9)	−0.0477 (11)	−0.0361 (9)
O2	0.0957 (12)	0.1031 (13)	0.0630 (10)	0.0266 (10)	−0.0349 (9)	−0.0180 (9)
O3	0.0636 (9)	0.0718 (9)	0.0633 (9)	0.0197 (7)	−0.0188 (7)	−0.0218 (7)
O1A	0.0576 (8)	0.0869 (11)	0.0582 (9)	0.0040 (8)	0.0064 (7)	0.0023 (8)
O2A	0.0614 (8)	0.0535 (8)	0.0740 (10)	0.0239 (6)	−0.0188 (7)	−0.0146 (7)
O3A	0.0659 (8)	0.0636 (8)	0.0467 (7)	0.0129 (7)	−0.0236 (6)	−0.0106 (6)

### Geometric parameters (Å, º)

**Table:**

C1—N1	1.460 (2)	C1A—C9A	1.514 (2)
C1—C9	1.511 (3)	C1A—C2A	1.546 (2)
C1—C2	1.545 (3)	C1A—H1A	0.9800
C1—H1	0.9800	C2A—C8A	1.500 (2)
C2—C8	1.497 (3)	C2A—C3A	1.538 (3)
C2—C3	1.531 (3)	C2A—H2A	0.9800
C2—H2	0.9800	C3A—C4A	1.525 (3)
C3—C4	1.516 (3)	C3A—H3A1	0.9700
C3—H3A	0.9700	C3A—H3A2	0.9700
C3—H3B	0.9700	C4A—C5A	1.526 (3)
C4—C5	1.515 (3)	C4A—H4A1	0.9700
C4—H4A	0.9700	C4A—H4A2	0.9700
C4—H4B	0.9700	C5A—C6A	1.540 (3)
C5—C6	1.531 (3)	C5A—H5A1	0.9700
C5—H5A	0.9700	C5A—H5A2	0.9700
C5—H5B	0.9700	C6A—C8A	1.505 (2)
C6—C8	1.498 (3)	C6A—C7A	1.552 (2)
C6—C7	1.549 (3)	C6A—H6A	0.9800
C6—H6	0.9800	C7A—N1A	1.466 (2)
C7—N1	1.464 (2)	C7A—C17A	1.513 (2)
C7—C17	1.509 (2)	C7A—H7A	0.9800
C7—H7	0.9800	C8A—O1A	1.210 (2)
C8—O1	1.210 (2)	C9A—C10A	1.370 (3)
C9—C10	1.364 (3)	C9A—C14A	1.403 (2)
C9—C14	1.409 (3)	C10A—C11A	1.391 (3)
C10—C11	1.390 (3)	C10A—H10A	0.9300
C10—H10	0.9300	C11A—C12A	1.368 (3)
C11—C12	1.380 (4)	C11A—H11A	0.9300
C11—H11	0.9300	C12A—C13A	1.376 (3)
C12—C13	1.359 (4)	C12A—H12A	0.9300
C12—H12	0.9300	C13A—C14A	1.385 (3)
C13—C14	1.378 (3)	C13A—H13A	0.9300
C13—H13	0.9300	C14A—O2A	1.362 (2)
C14—O2	1.356 (3)	C15A—O2A	1.420 (2)
C15—O2	1.416 (3)	C15A—C16A	1.485 (3)
C15—C16	1.489 (4)	C15A—H15C	0.9700
C15—H15A	0.9700	C15A—H15D	0.9700
C15—H15B	0.9700	C16A—H16D	0.9600
C16—H16A	0.9600	C16A—H16E	0.9600
C16—H16B	0.9600	C16A—H16F	0.9600
C16—H16C	0.9600	C17A—C22A	1.376 (2)
C17—C22	1.376 (3)	C17A—C18A	1.402 (2)
C17—C18	1.402 (2)	C18A—O3A	1.362 (2)
C18—O3	1.359 (2)	C18A—C19A	1.385 (2)
C18—C19	1.385 (3)	C19A—C20A	1.369 (3)
C19—C20	1.374 (3)	C19A—H19A	0.9300
C19—H19	0.9300	C20A—C21A	1.373 (3)
C20—C21	1.370 (3)	C20A—H20A	0.9300
C20—H20	0.9300	C21A—C22A	1.386 (3)
C21—C22	1.386 (3)	C21A—H21A	0.9300
C21—H21	0.9300	C22A—H22A	0.9300
C22—H22	0.9300	C23A—O3A	1.429 (2)
C23—O3	1.429 (2)	C23A—C24A	1.482 (3)
C23—C24	1.484 (3)	C23A—H23C	0.9700
C23—H23A	0.9700	C23A—H23D	0.9700
C23—H23B	0.9700	C24A—H24D	0.9600
C24—H24A	0.9600	C24A—H24E	0.9600
C24—H24B	0.9600	C24A—H24F	0.9600
C24—H24C	0.9600	N1—H1B	0.8600
C1A—N1A	1.460 (2)	N1A—H1A1	0.8600
			
N1—C1—C9	112.73 (15)	C2A—C1A—H1A	108.0
N1—C1—C2	109.67 (14)	C8A—C2A—C3A	108.26 (15)
C9—C1—C2	109.74 (14)	C8A—C2A—C1A	107.36 (13)
N1—C1—H1	108.2	C3A—C2A—C1A	115.16 (15)
C9—C1—H1	108.2	C8A—C2A—H2A	108.6
C2—C1—H1	108.2	C3A—C2A—H2A	108.6
C8—C2—C3	107.69 (16)	C1A—C2A—H2A	108.6
C8—C2—C1	107.31 (15)	C4A—C3A—C2A	114.66 (15)
C3—C2—C1	115.21 (16)	C4A—C3A—H3A1	108.6
C8—C2—H2	108.8	C2A—C3A—H3A1	108.6
C3—C2—H2	108.8	C4A—C3A—H3A2	108.6
C1—C2—H2	108.8	C2A—C3A—H3A2	108.6
C4—C3—C2	114.79 (16)	H3A1—C3A—H3A2	107.6
C4—C3—H3A	108.6	C3A—C4A—C5A	113.24 (17)
C2—C3—H3A	108.6	C3A—C4A—H4A1	108.9
C4—C3—H3B	108.6	C5A—C4A—H4A1	108.9
C2—C3—H3B	108.6	C3A—C4A—H4A2	108.9
H3A—C3—H3B	107.5	C5A—C4A—H4A2	108.9
C5—C4—C3	114.22 (18)	H4A1—C4A—H4A2	107.7
C5—C4—H4A	108.7	C4A—C5A—C6A	113.95 (15)
C3—C4—H4A	108.7	C4A—C5A—H5A1	108.8
C5—C4—H4B	108.7	C6A—C5A—H5A1	108.8
C3—C4—H4B	108.7	C4A—C5A—H5A2	108.8
H4A—C4—H4B	107.6	C6A—C5A—H5A2	108.8
C4—C5—C6	113.58 (16)	H5A1—C5A—H5A2	107.7
C4—C5—H5A	108.8	C8A—C6A—C5A	107.13 (15)
C6—C5—H5A	108.8	C8A—C6A—C7A	106.65 (14)
C4—C5—H5B	108.8	C5A—C6A—C7A	116.51 (15)
C6—C5—H5B	108.8	C8A—C6A—H6A	108.8
H5A—C5—H5B	107.7	C5A—C6A—H6A	108.8
C8—C6—C5	107.26 (16)	C7A—C6A—H6A	108.8
C8—C6—C7	108.30 (15)	N1A—C7A—C17A	110.19 (13)
C5—C6—C7	115.01 (15)	N1A—C7A—C6A	110.42 (13)
C8—C6—H6	108.7	C17A—C7A—C6A	113.17 (13)
C5—C6—H6	108.7	N1A—C7A—H7A	107.6
C7—C6—H6	108.7	C17A—C7A—H7A	107.6
N1—C7—C17	111.45 (14)	C6A—C7A—H7A	107.6
N1—C7—C6	110.49 (14)	O1A—C8A—C2A	123.74 (16)
C17—C7—C6	110.87 (14)	O1A—C8A—C6A	124.56 (16)
N1—C7—H7	108.0	C2A—C8A—C6A	111.70 (15)
C17—C7—H7	108.0	C10A—C9A—C14A	118.50 (16)
C6—C7—H7	108.0	C10A—C9A—C1A	122.21 (15)
O1—C8—C2	123.55 (19)	C14A—C9A—C1A	119.28 (16)
O1—C8—C6	124.69 (19)	C9A—C10A—C11A	121.32 (19)
C2—C8—C6	111.73 (15)	C9A—C10A—H10A	119.3
C10—C9—C14	118.76 (19)	C11A—C10A—H10A	119.3
C10—C9—C1	122.92 (18)	C12A—C11A—C10A	119.4 (2)
C14—C9—C1	118.23 (18)	C12A—C11A—H11A	120.3
C9—C10—C11	121.2 (2)	C10A—C11A—H11A	120.3
C9—C10—H10	119.4	C11A—C12A—C13A	120.68 (19)
C11—C10—H10	119.4	C11A—C12A—H12A	119.7
C12—C11—C10	118.8 (3)	C13A—C12A—H12A	119.7
C12—C11—H11	120.6	C12A—C13A—C14A	119.83 (19)
C10—C11—H11	120.6	C12A—C13A—H13A	120.1
C13—C12—C11	121.3 (2)	C14A—C13A—H13A	120.1
C13—C12—H12	119.3	O2A—C14A—C13A	124.40 (17)
C11—C12—H12	119.3	O2A—C14A—C9A	115.37 (15)
C12—C13—C14	119.9 (2)	C13A—C14A—C9A	120.23 (19)
C12—C13—H13	120.1	O2A—C15A—C16A	107.61 (18)
C14—C13—H13	120.1	O2A—C15A—H15C	110.2
O2—C14—C13	124.5 (2)	C16A—C15A—H15C	110.2
O2—C14—C9	115.44 (18)	O2A—C15A—H15D	110.2
C13—C14—C9	120.1 (2)	C16A—C15A—H15D	110.2
O2—C15—C16	107.1 (2)	H15C—C15A—H15D	108.5
O2—C15—H15A	110.3	C15A—C16A—H16D	109.5
C16—C15—H15A	110.3	C15A—C16A—H16E	109.5
O2—C15—H15B	110.3	H16D—C16A—H16E	109.5
C16—C15—H15B	110.3	C15A—C16A—H16F	109.5
H15A—C15—H15B	108.6	H16D—C16A—H16F	109.5
C15—C16—H16A	109.5	H16E—C16A—H16F	109.5
C15—C16—H16B	109.5	C22A—C17A—C18A	118.05 (15)
H16A—C16—H16B	109.5	C22A—C17A—C7A	122.34 (15)
C15—C16—H16C	109.5	C18A—C17A—C7A	119.57 (15)
H16A—C16—H16C	109.5	O3A—C18A—C19A	124.10 (16)
H16B—C16—H16C	109.5	O3A—C18A—C17A	115.66 (14)
C22—C17—C18	118.06 (17)	C19A—C18A—C17A	120.25 (17)
C22—C17—C7	122.51 (16)	C20A—C19A—C18A	119.99 (18)
C18—C17—C7	119.43 (16)	C20A—C19A—H19A	120.0
O3—C18—C19	124.35 (17)	C18A—C19A—H19A	120.0
O3—C18—C17	115.60 (16)	C19A—C20A—C21A	120.89 (18)
C19—C18—C17	120.05 (18)	C19A—C20A—H20A	119.6
C20—C19—C18	120.28 (19)	C21A—C20A—H20A	119.6
C20—C19—H19	119.9	C20A—C21A—C22A	118.97 (19)
C18—C19—H19	119.9	C20A—C21A—H21A	120.5
C21—C20—C19	120.57 (19)	C22A—C21A—H21A	120.5
C21—C20—H20	119.7	C17A—C22A—C21A	121.78 (17)
C19—C20—H20	119.7	C17A—C22A—H22A	119.1
C20—C21—C22	119.1 (2)	C21A—C22A—H22A	119.1
C20—C21—H21	120.4	O3A—C23A—C24A	107.22 (18)
C22—C21—H21	120.4	O3A—C23A—H23C	110.3
C17—C22—C21	121.93 (19)	C24A—C23A—H23C	110.3
C17—C22—H22	119.0	O3A—C23A—H23D	110.3
C21—C22—H22	119.0	C24A—C23A—H23D	110.3
O3—C23—C24	107.78 (19)	H23C—C23A—H23D	108.5
O3—C23—H23A	110.2	C23A—C24A—H24D	109.5
C24—C23—H23A	110.2	C23A—C24A—H24E	109.5
O3—C23—H23B	110.2	H24D—C24A—H24E	109.5
C24—C23—H23B	110.2	C23A—C24A—H24F	109.5
H23A—C23—H23B	108.5	H24D—C24A—H24F	109.5
C23—C24—H24A	109.5	H24E—C24A—H24F	109.5
C23—C24—H24B	109.5	C1—N1—C7	112.51 (14)
H24A—C24—H24B	109.5	C1—N1—H1B	123.7
C23—C24—H24C	109.5	C7—N1—H1B	123.7
H24A—C24—H24C	109.5	C1A—N1A—C7A	112.04 (13)
H24B—C24—H24C	109.5	C1A—N1A—H1A1	124.0
N1A—C1A—C9A	111.76 (14)	C7A—N1A—H1A1	124.0
N1A—C1A—C2A	109.47 (13)	C14—O2—C15	120.69 (19)
C9A—C1A—C2A	111.38 (13)	C18—O3—C23	119.19 (15)
N1A—C1A—H1A	108.0	C14A—O2A—C15A	119.74 (16)
C9A—C1A—H1A	108.0	C18A—O3A—C23A	119.32 (15)
			
N1—C1—C2—C8	−59.41 (19)	C8A—C6A—C7A—N1A	57.59 (17)
C9—C1—C2—C8	176.24 (15)	C5A—C6A—C7A—N1A	−61.93 (18)
N1—C1—C2—C3	60.5 (2)	C8A—C6A—C7A—C17A	−178.36 (14)
C9—C1—C2—C3	−63.9 (2)	C5A—C6A—C7A—C17A	62.12 (19)
C8—C2—C3—C4	50.3 (2)	C3A—C2A—C8A—O1A	116.7 (2)
C1—C2—C3—C4	−69.4 (2)	C1A—C2A—C8A—O1A	−118.4 (2)
C2—C3—C4—C5	−41.6 (2)	C3A—C2A—C8A—C6A	−63.49 (19)
C3—C4—C5—C6	43.3 (2)	C1A—C2A—C8A—C6A	61.42 (19)
C4—C5—C6—C8	−53.8 (2)	C5A—C6A—C8A—O1A	−115.0 (2)
C4—C5—C6—C7	66.7 (2)	C7A—C6A—C8A—O1A	119.6 (2)
C8—C6—C7—N1	55.34 (19)	C5A—C6A—C8A—C2A	65.14 (19)
C5—C6—C7—N1	−64.60 (19)	C7A—C6A—C8A—C2A	−60.28 (18)
C8—C6—C7—C17	179.42 (15)	N1A—C1A—C9A—C10A	−22.4 (2)
C5—C6—C7—C17	59.5 (2)	C2A—C1A—C9A—C10A	100.38 (19)
C3—C2—C8—O1	114.5 (2)	N1A—C1A—C9A—C14A	159.10 (15)
C1—C2—C8—O1	−120.9 (2)	C2A—C1A—C9A—C14A	−78.10 (19)
C3—C2—C8—C6	−63.8 (2)	C14A—C9A—C10A—C11A	0.3 (3)
C1—C2—C8—C6	60.8 (2)	C1A—C9A—C10A—C11A	−178.19 (18)
C5—C6—C8—O1	−112.5 (2)	C9A—C10A—C11A—C12A	0.6 (3)
C7—C6—C8—O1	122.8 (2)	C10A—C11A—C12A—C13A	−1.0 (3)
C5—C6—C8—C2	65.9 (2)	C11A—C12A—C13A—C14A	0.5 (3)
C7—C6—C8—C2	−58.8 (2)	C12A—C13A—C14A—O2A	−179.41 (18)
N1—C1—C9—C10	−23.5 (3)	C12A—C13A—C14A—C9A	0.4 (3)
C2—C1—C9—C10	99.1 (2)	C10A—C9A—C14A—O2A	179.03 (16)
N1—C1—C9—C14	160.12 (16)	C1A—C9A—C14A—O2A	−2.4 (2)
C2—C1—C9—C14	−77.3 (2)	C10A—C9A—C14A—C13A	−0.8 (3)
C14—C9—C10—C11	0.5 (3)	C1A—C9A—C14A—C13A	177.77 (16)
C1—C9—C10—C11	−175.9 (2)	N1A—C7A—C17A—C22A	37.4 (2)
C9—C10—C11—C12	−0.3 (4)	C6A—C7A—C17A—C22A	−86.8 (2)
C10—C11—C12—C13	0.4 (4)	N1A—C7A—C17A—C18A	−140.27 (15)
C11—C12—C13—C14	−0.7 (4)	C6A—C7A—C17A—C18A	95.57 (18)
C12—C13—C14—O2	−179.9 (2)	C22A—C17A—C18A—O3A	177.77 (15)
C12—C13—C14—C9	1.0 (3)	C7A—C17A—C18A—O3A	−4.5 (2)
C10—C9—C14—O2	180.0 (2)	C22A—C17A—C18A—C19A	−2.6 (2)
C1—C9—C14—O2	−3.5 (3)	C7A—C17A—C18A—C19A	175.13 (15)
C10—C9—C14—C13	−0.8 (3)	O3A—C18A—C19A—C20A	−177.73 (17)
C1—C9—C14—C13	175.74 (18)	C17A—C18A—C19A—C20A	2.7 (3)
N1—C7—C17—C22	24.0 (2)	C18A—C19A—C20A—C21A	−0.4 (3)
C6—C7—C17—C22	−99.5 (2)	C19A—C20A—C21A—C22A	−1.9 (3)
N1—C7—C17—C18	−156.54 (16)	C18A—C17A—C22A—C21A	0.3 (3)
C6—C7—C17—C18	79.9 (2)	C7A—C17A—C22A—C21A	−177.36 (18)
C22—C17—C18—O3	179.19 (17)	C20A—C21A—C22A—C17A	1.9 (3)
C7—C17—C18—O3	−0.3 (2)	C9—C1—N1—C7	−177.56 (15)
C22—C17—C18—C19	−0.8 (3)	C2—C1—N1—C7	59.85 (19)
C7—C17—C18—C19	179.73 (17)	C17—C7—N1—C1	178.51 (14)
O3—C18—C19—C20	−179.18 (18)	C6—C7—N1—C1	−57.74 (19)
C17—C18—C19—C20	0.8 (3)	C9A—C1A—N1A—C7A	−176.10 (13)
C18—C19—C20—C21	−0.1 (3)	C2A—C1A—N1A—C7A	60.01 (17)
C19—C20—C21—C22	−0.6 (3)	C17A—C7A—N1A—C1A	174.44 (13)
C18—C17—C22—C21	0.1 (3)	C6A—C7A—N1A—C1A	−59.81 (17)
C7—C17—C22—C21	179.55 (18)	C13—C14—O2—C15	−9.5 (3)
C20—C21—C22—C17	0.6 (3)	C9—C14—O2—C15	169.7 (2)
N1A—C1A—C2A—C8A	−58.99 (18)	C16—C15—O2—C14	−174.7 (2)
C9A—C1A—C2A—C8A	176.90 (14)	C19—C18—O3—C23	0.6 (3)
N1A—C1A—C2A—C3A	61.64 (18)	C17—C18—O3—C23	−179.40 (17)
C9A—C1A—C2A—C3A	−62.47 (18)	C24—C23—O3—C18	171.1 (2)
C8A—C2A—C3A—C4A	50.9 (2)	C13A—C14A—O2A—C15A	−6.7 (3)
C1A—C2A—C3A—C4A	−69.3 (2)	C9A—C14A—O2A—C15A	173.51 (16)
C2A—C3A—C4A—C5A	−42.5 (2)	C16A—C15A—O2A—C14A	−176.14 (18)
C3A—C4A—C5A—C6A	44.4 (2)	C19A—C18A—O3A—C23A	−4.5 (3)
C4A—C5A—C6A—C8A	−54.4 (2)	C17A—C18A—O3A—C23A	175.13 (16)
C4A—C5A—C6A—C7A	64.9 (2)	C24A—C23A—O3A—C18A	178.68 (18)

### Hydrogen-bond geometry (Å, º)

**Table:**

*D*—H···*A*	*D*—H	H···*A*	*D*···*A*	*D*—H···*A*
C23—H23*A*···O1*A*	0.97	2.42	3.311 (3)	153
C23*A*—H23*C*···O1	0.97	2.43	3.297 (3)	149
